# Supplementary material for: Spatial and temporal organization of RecA in the Escherichia coli DNA-damage response
Source: eLife. 2019 Feb 5;8:e42761. doi: 10.7554/eLife.42761 (PMC6363387; doi:10.7554/eLife.42761)
Supplement: Supplementary file 1. — Supplemental Table 1: Description of plasmids used in this study. Supplemental Table 2: List of strains used in this study. Supplemental Table 3: List and sequences of primers used in this study. Supplemental Table 4: Sequences of plasmids used in this study. Supplemental Table 5: Sequences of inserts used for cloning. [file elife-42761-supp1.docx]

# Supplemental table 1: Description of plasmids used in this study

|  | **Plasmid name** | **Plasmid description in text** | **Parent vector** | **Description** | **Source** |
| --- | --- | --- | --- | --- | --- |
| 1 | pHG001 | pJM1071 | pGB1 | low copy vector with constitutive *recA* promoter and native *recA* RBS | Woodgate lab (Churchward, Belin, & Nagamine, 1984) |
| 2 | pHG002 | pConst-*mKate2* | pJM1071 | *mKate2* cloned between *Nde*I/*Xba*I and expressed from native *recA* RBS | This work |
| 3 | pHG004 | pG353C-*mKate2* | pJM1071 | *mKate2* cloned between *Nde*I/*Xba*I and expressed from mutant *recA* RBS | This work |
| 4 | pHG009 |  | pBAD-myc-HisB |  | Poolman Lab |
| 5 | pHG043 | pBAD-S | pBAD-myc-HisB | FRT-Kan-FRT from pEAW507 was cloned into pBAD-myc-HisB between *Nco*I/*Sal*I | This work |
| 6 | pHG064 | pBAD-*YPet-mcI* | pHG043 | *YPet-mcI* cloned into pBAD-S between *Not*I/*Apa*I | This work |
| 7 | pHG074 | pBAD-S-*mcI* | pHG043 | *mcI* cloned into pHG043 between *Not*I/*Apa*I | This work |
| 8 | pHG076 | pBAD-*PAmCherry-mcI* | pHG043 | *Xba*I digest to remove FRT-Kan-FRT cassette from pHG080 | This work |
| 9 | pHG080 | pBAD-S-*PAmCherry-mcI* | pHG043 | *PAmCherry1* cloned between *Not*I/*Pst*I in pHG064 |  |
| 10 | pHG081 | pBAD-*mcI* | pHG043 | *Xba*I digest to remove FRT-Kan-FRT cassette from pHG074 |  |
| 11 | pUA66-gfp | *gfp* |  | *gfpmut2* | Alon Lab |
| 12 | pUA139-sulAp-gfp | *sulAp-gfp* |  | *gfpmut2* under *sulA* promoter | Alon Lab |
| 13 | pUA66-recAp-gfp | pRecAp-gfp |  | *gfpmut2* under *recA* promoter | Alon lab |
| 14 | pHG087 | pBAD-*YPet-mcI* | pHG064 | *Xba*I digest to remove FRT-Kan-FRT cassette | This work |
| 15 | pHG102 | pETMCSI-*mcI* | pET MCSI | *mcI* cloned into pETMCSIII between *Nde*I/*Eco*RI | This work |
| 16 | pHG101 | pETMCSI- *YPet-mcI* | pET MCSI | *YPet-mcI* cloned into pETMCSIII between *Nde*I/*Eco*RI | This work |
| 17 | pHG115 | pND706-*PAmCherry(R202H)-mcI* | pND706 | *PAmCherry-mcI* cloned into pETMCSIII between *Nde*I/*Eco*RI | This work |
| 18 | pHG133 | pG353C-*recA* | pJM1071 | *recA* cloned in pHG004 between *Nde*I/*Xba*I | This work |
| 19 | pHG134 | pConst-*recA* | pJM1071 | *recA* cloned in pJM1071 between *Nde*I/*Xba*I | This work |
| 20 | pEAW846 | pBAD-*dinI* | pBAD | *dinI* | This work |

# Supplemental table 2: List of strains used in this study

| **Strain Name** | **Parent strain** | **Genotype** | **Plasmids** | **Description** | **Used in Figure** | **Source** |
| --- | --- | --- | --- | --- | --- | --- |
| HG001 | MG1655 |  |  |  |  | Cox lab |
| HG257 | MG1655 | MG1655 | gfp, pBAD-myc-HisB | *gfp* + *mcI* | 3 | This work |
| HG258 | MG1655 | MG1655 | pUA139-*sulAp-gfp*, pBAD-myc-HisB | *sulAp-gfp* + pBAD | 3 | This work |
| HG252 | MG1655 | MG1655 | pUA66-*gfp*, pHG081 | g*fp* + *mcI* | 3 | This work |
| HG253 | MG1655 | MG1655 | pUA139-*sulAp-gfp*, pHG081 | *sulAp-gfp* + *mcI* | 3 | This work |
| HG284 | MG1655 | MG1655 | pUA66-*gfp* + pHG076 | *gfp* + *PAmCherry-mcI* | 3 | This work |
| HG285 | MG1655 | MG1655 | pUA66-s*ulAp-gfp*, pHG076 | *sulAp-gfp* + *PAmCherry-mcI* | 3 | This work |
| HG260 | MG1655 | MG1655 | pUA66-*recAp-gfp*, pBAD-myc-HisB | *recA-gfp* + pBAD | 1 | This work |
| RW1572 | MG1655 | *dnaQ*::Ypet *lexA3*(Ind^-^) *malB*::Tn*9* |  |  |  | This work |
| AR172 | MG1655 | *dnaQ*::Ypet |  |  |  | Cox lab (Robinson et al., 2015) |
| HG267 | MG1655 | *dnaQ*::Ypet | pHG076 |  | 4 | This work |
| HG311 | MG1655 | *dnaQ*::ypet *lexA3*(Ind^-^) *malB*::Tn*9* | pHG076 |  | 4 | This work |
| HG242 | DH5α | DH5α | pHG064 |  | 5 | This work |
| HG143 | MG1655 | MG1655 | pHG087 | pBAD-*YPet-mcI* | 5 | This work |
| HG297 | BL21(DE3) *recA*^-^ | BL21(DE3) *recA*^-^ | pHG100 | IPTG inducible expression plasmid encoding for untagged *mcI* | 2 | This work |
| HG298 | BL21(DE3) *recA*^-^ | BL21(DE3) *recA*^-^ | pHG101 | IPTG inducible expression plasmid encoding for untagged *YPet-mcI* | 2 | This work |
| HG195 | MG1655 | *recA-gfp* |  |  | 1, 6 | Weibel lab |
| HG411 | HG195 | *recA-gfp* | pHG134 |  | 6 | This work |
| HG412 | HG195 | *recA-gfp* | pHG134, pHG081 |  |  | This work |
| HG406 | HG195 | *recA-gfp* | pHG133 |  | 6 | This work |
| HG408 | HG195 | *recA-gfp* | pHG133, pHG081 |  |  | This work |
| HG446 | MG1655 |  | pHG134, pHG064 |  | 6 | This work |
| HG465 | MG1655 |  | pHG134 |  | 6 | This work |
| HG019 | MG1655 | Δ*uvrD* |  |  |  | This work |
| HG235 | HG019 | Δ*uvrD* | pHG064 |  | Figure 5 – figure supplement 1 | This work |
| HH020 | MG1655 | Δ*recA* |  |  | 6 | This work |
| EAW428 | MG1655 | *recA-gfp* |  | P_wt_-*recA-gfp*; *recA-gfp* fusion with wild-type RecA promoter | 6 | This work |
| EAW767 | EAW428 | *recA-gfp* Δ*dinI* |  |  | 6 | This work |
| HG472 | EAW767 |  | pEAW846 |  | Figure 6 – figure supplement 1 | This work |
| HG227 | MG1655 |  |  | pConst-*mKate2* | Figure 6 – figure supplement 1 | This work |
| HG004 | MG1655 |  | pHG004 | pG353C-*mKate2* | Figure 6 – figure supplement 1 | This work |
| HG116 | MG1655 |  | pBAD-myc-HisB |  | Figure 4 – figure supplement 1 | This work |

# Supplemental table 3: List of primers used in this study

| **Primers** | **Sequence** |
| --- | --- |
| NtermFusion_FW | GAGGAATTAA CCATGG CGGCCG CATATG TCG GCT GGC TCC GCT GCA GGT TCT GGC GAA TTC TAA GGGCCC ccctttcgtcttcaagaattc |
| NtermFusion_R | GATGATGATGGTCGACGGC ggccacgatgcgtccggcgta |
| mcI mCardinal_FW | ATGACAGGAGGCGGCCGCATCAT ATGGTGAGCAAGGGCGAG |
| mcI mCardinal_R | CCGCCGCCACCTGCAGCGAGCTC CTTGTACAGCTCGTCCATGCC |
| JM1071_mK2_g353c_S | cccggcatgacaggactaacatatggtgagc |
| JM1071_mK2_g353c_AS | gctcaccatatgttagtcctgtcatgccggg |

# Supplemental table 4: Sequences of plasmids used in this study

| **Plasmid name** | **Sequence** |
| --- | --- |
| pBAD-S  (pHG043) | CCCTAACTGCTGGCGGAAAAGATGTGACAGACGCGACGGCGACAAGCAAACATGCTGTGCGACGCTGGCGATATCAAAATTGCTGTCTGCCAGGTGATCGCTGATGTACTGACAAGCCTCGCGTACCCGATTATCCATCGGTGGATGGAGCGACTCGTTAATCGCTTCCATGCGCCGCAGTAACAATTGCTCAAGCAGATTTATCGCCAGCAGCTCCGAATAGCGCCCTTCCCCTTGCCCGGCGTTAATGATTTGCCCAAACAGGTCGCTGAAATGCGGCTGGTGCGCTTCATCCGGGCGAAAGAACCCCGTATTGGCAAATATTGACGGCCAGTTAAGCCATTCATGCCAGTAGGCGCGCGGACGAAAGTAAACCCACTGGTGATACCATTCGCGAGCCTCCGGATGACGACCGTAGTGATGAATCTCTCCTGGCGGGAACAGCAAAATATCACCCGGTCGGCAAACAAATTCTCGTCCCTGATTTTTCACCACCCCCTGACCGCGAATGGTGAGATTGAGAATATAACCTTTCATTCCCAGCGGTCGGTCGATAAAAAAATCGAGATAACCGTTGGCCTCAATCGGCGTTAAACCCGCCACCAGATGGGCATTAAACGAGTATCCCGGCAGCAGGGGATCATTTTGCGCTTCAGCCATACTTTTCATACTCCCGCCATTCAGAGAAGAAACCAATTGTCCATATTGCATCAGACATTGCCGTCACTGCGTCTTTTACTGGCTCTTCTCGCTAACCAAACCGGTAACCCCGCTTATTAAAAGCATTCTGTAACAAAGCGGGACCAAAGCCATGACAAAAACGCGTAACAAAAGTGTCTATAATCACGGCAGAAAAGTCCACATTGATTATTTGCACGGCGTCACACTTTGCTATGCCATAGCATTTTTATCCATAAGATTAGCGGATCCTACCTGACGCTTTTTATCGCAACTCTCTACTGTTTCTCCATACCCGTTTTTTGGGCTAACAGGAGGAATTAACCATGGCGGCCGCATATGTCGGCTGGCTCCGCTGCAGGTTCTGGCGAATTCTAAGGGCCCCCCTTTCGTCTTCAAGAATTCGAAGTTCCTATAGTTTCTAGAGAATAGGAACTTCgatctttagaaaaactcatcgagcatcaaatgaaactgcaatttattcatatcaggattatcaataccatatttttgaaaaagccgtttctgtaatgaaggagaaaactcaccgaggcagttccataggatggcaagatcctggtatcggtctgcgattccgactcgtccaacatcaatacaacctattaatttcccctcgtcaaaaataaggttatcaagtgagaaatcaccatgagtgacgactgaatccggtgagaatggcaaaagcttatgcatttctttccagacttgttcaacaggccagccattacgctcgtcatcaaaatcactcgcatcaaccaaaccgttattcattcgtgattgcgcctgagcgagacgaaatacgcgatcgctgttaaaaggacaattacaaacaggaatcgaatgcaaccggcgcaggaacactgccagcgcatcaacaatattttcacctgaatcaggatattcttctaatacctggaatgctgttttcccggggatcgcagtggtgagtaaccatgcatcatcaggagtacggataaaatgcttgatggtcggaagaggcataaattccgtcagccagtttagtctgaccatctcatctgtaacatcattggcaacgctacctttgccatgtttcagaaacaactctggcgcatcgggcttcccatacaatcgatagattgtcgcacctgattgcccgacattatcgcgagcccatttatacccatataaatcagcatccatgttggaatttaatcgcggcctcgagcaagacgtttcccgttgaatatggctcataacaccccttgtattactgtttatgtaagcagacagttttattgttcatgatgatatatttttatcttgtgcaatgtaacatcagagattttgagacacaacgtggctttccccccccccccgatccccgggtaccgagctcgaatttcgaccaattcGAAGTTCCTATACTTTCTAGAGAATAGGAACTTCcgggatcctcTACGCCGGACGCATCGTGGCCGCCGTCGACCATCATCATCATCATCATTGAGTTTAAACGGTCTCCAGCTTGGCTGTTTTGGCGGATGAGAGAAGATTTTCAGCCTGATACAGATTAAATCAGAACGCAGAAGCGGTCTGATAAAACAGAATTTGCCTGGCGGCAGTAGCGCGGTGGTCCCACCTGACCCCATGCCGAACTCAGAAGTGAAACGCCGTAGCGCCGATGGTAGTGTGGGGTCTCCCCATGCGAGAGTAGGGAACTGCCAGGCATCAAATAAAACGAAAGGCTCAGTCGAAAGACTGGGCCTTTCGTTTTATCTGTTGTTTGTCGGTGAACGCTCTCCTGAGTAGGACAAATCCGCCGGGAGCGGATTTGAACGTTGCGAAGCAACGGCCCGGAGGGTGGCGGGCAGGACGCCCGCCATAAACTGCCAGGCATCAAATTAAGCAGAAGGCCATCCTGACGGATGGCCTTTTTGCGTTTCTACAAACTCTTTTGTTTATTTTTCTAAATACATTCAAATATGTATCCGCTCATGAGACAATAACCCTGATAAATGCTTCAATAATATTGAAAAAGGAAGAGTATGAGTATTCAACATTTCCGTGTCGCCCTTATTCCCTTTTTTGCGGCATTTTGCCTTCCTGTTTTTGCTCACCCAGAAACGCTGGTGAAAGTAAAAGATGCTGAAGATCAGTTGGGTGCACGAGTGGGTTACATCGAACTGGATCTCAACAGCGGTAAGATCCTTGAGAGTTTTCGCCCCGAAGAACGTTTTCCAATGATGAGCACTTTTAAAGTTCTGCTATGTGGCGCGGTATTATCCCGTGTTGACGCCGGGCAAGAGCAACTCGGTCGCCGCATACACTATTCTCAGAATGACTTGGTTGAGTACTCACCAGTCACAGAAAAGCATCTTACGGATGGCATGACAGTAAGAGAATTATGCAGTGCTGCCATAACCATGAGTGATAACACTGCGGCCAACTTACTTCTGACAACGATCGGAGGACCGAAGGAGCTAACCGCTTTTTTGCACAACATGGGGGATCATGTAACTCGCCTTGATCGTTGGGAACCGGAGCTGAATGAAGCCATACCAAACGACGAGCGTGACACCACGATGCCTGTAGCAATGGCAACAACGTTGCGCAAACTATTAACTGGCGAACTACTTACTCTAGCTTCCCGGCAACAATTAATAGACTGGATGGAGGCGGATAAAGTTGCAGGACCACTTCTGCGCTCGGCCCTTCCGGCTGGCTGGTTTATTGCTGATAAATCTGGAGCCGGTGAGCGTGGGTCTCGCGGTATCATTGCAGCACTGGGGCCAGATGGTAAGCCCTCCCGTATCGTAGTTATCTACACGACGGGGAGTCAGGCAACTATGGATGAACGAAATAGACAGATCGCTGAGATAGGTGCCTCACTGATTAAGCATTGGTAACTGTCAGACCAAGTTTACTCATATATACTTTAGATTGATTTAAAACTTCATTTTTAATTTAAAAGGATCTAGGTGAAGATCCTTTTTGATAATCTCATGACCAAAATCCCTTAACGTGAGTTTTCGTTCCACTGAGCGTCAGACCCCGTAGAAAAGATCAAAGGATCTTCTTGAGATCCTTTTTTTCTGCGCGTAATCTGCTGCTTGCAAACAAAAAAACCACCGCTACCAGCGGTGGTTTGTTTGCCGGATCAAGAGCTACCAACTCTTTTTCCGAAGGTAACTGGCTTCAGCAGAGCGCAGATACCAAATACTGTCCTTCTAGTGTAGCCGTAGTTAGGCCACCACTTCAAGAACTCTGTAGCACCGCCTACATACCTCGCTCTGCTAATCCTGTTACCAGTGGCTGCTGCCAGTGGCGATAAGTCGTGTCTTACCGGGTTGGACTCAAGACGATAGTTACCGGATAAGGCGCAGCGGTCGGGCTGAACGGGGGGTTCGTGCACACAGCCCAGCTTGGAGCGAACGACCTACACCGAACTGAGATACCTACAGCGTGAGCTATGAGAAAGCGCCACGCTTCCCGAAGGGAGAAAGGCGGACAGGTATCCGGTAAGCGGCAGGGTCGGAACAGGAGAGCGCACGAGGGAGCTTCCAGGGGGAAACGCCTGGTATCTTTATAGTCCTGTCGGGTTTCGCCACCTCTGACTTGAGCGTCGATTTTTGTGATGCTCGTCAGGGGGGCGGAGCCTATGGAAAAACGCCAGCAACGCGGCCTTTTTACGGTTCCTGGCCTTTTGCTGGCCTTTTGCTCACATGTTCTTTCCTGCGTTATCCCCTGATTCTGTGGATAACCGTATTACCGCCTTTGAGTGAGCTGATACCGCTCGCCGCAGCCGAACGACCGAGCGCAGCGAGTCAGTGAGCGAGGAAGCGGAAGAGCGCCTGATGCGGTATTTTCTCCTTACGCATCTGTGCGGTATTTCACACCGCATATGGTGCACTCTCAGTACAATCTGCTCTGATGCCGCATAGTTAAGCCAGTATACACTCCGCTATCGCTACGTGACTGGGTCATGGCTGCGCCCCGACACCCGCCAACACCCGCTGACGCGCCCTGACGGGCTTGTCTGCTCCCGGCATCCGCTTACAGACAAGCTGTGACCGTCTCCGGGAGCTGCATGTGTCAGAGGTTTTCACCGTCATCACCGAAACGCGCGAGGCAGCAGATCAATTCGCGCGCGAAGGCGAAGCGGCATGCATAATGTGCCTGTCAAATGGACGAAGCAGGGATTCTGCAAACCCTATGCTACTCCGTCAAGCCGTCAATTGTCTGATTCGTTACCAATTATGACAACTTGACGGCTACATCATTCACTTTTTCTTCACAACCGGCACGGAACTCGCTCGGGCTGGCCCCGGTGCATTTTTTAAATACCCGCGAGAAATAGAGTTGATCGTCAAAACCAACATTGCGACCGACGGTGGCGATAGGCATCCGGGTGGTGCTCAAAAGCAGCTTCGCCTGGCTGATACGTTGGTCCTCGCGCCAGCTTAAGACGCTAAT |
| pJM1071 | AAGCTGGAAGATCTTCCCTGGCACGACAGGTTTCCCGACTGGAAAGCGGGCAGTGAGCGCAACGCAATTAATGTGAGTTAGCTCACTCATTAGGCACCCCAGGCTTTACACTTTATGCTTCCGGCTCGTATGTTGTGTGGAATTGTGAGCGGATAACAATTTCACACAGGAAACAGCTATGACCATGATTACGCCAAGCGCGCAATTAACCCTCACTAAAGGGAACAAAAGCTGGGTACCGGGCCCCCCCTCGAGGTCGACTTTCTACAAAACACTTGATACTGTATGAGCATGCAGTATAATTGCTTCAACAGAACATATTGACTATCCGGTATTACCCGGCATGACAGGAGTAACATATGGAATTCCTGCAGCCCGGGGGATCCACTAGTTCTAGAGCGGCCGCCACCGCGGTGGAGCTCCAATTCGCCCTATAGTGAGTCGTATTACGCGCGCTCACTGGCCGTCGTTTTACAACGTCGTGACTGGGAAAACCCTGGCGTTACCCAACTTAATCGCCTTGCAGCACATCCCCCTTTCGCCAGCTGGCGTAATAGCGAAGAGGCCCGCACCGATCGCCCTTCCCAACAGTTGCGCAGCCTGAATGGCGAATGGGACGCGCCCTGTAGCGGCGCATTAAGCGCGGCGGGTGTGGTGGTTACGCGCAGCGTGACCGCTACACTTGCCAGCGCCCTAGCGCCCGCTCCTTTCGCTTTCTTCCCTTCCTTTCTCGCCACGTTCGCCGGAAGATCTTCCAATTCCCGACAGTAAGACGGGTAAGCCTGTTGATGATACCGCTGCCTTACTGGGTGCATTAGCCAGTCTGAATGACCTGTCACGGGATAATCCGAAGTGGTCAGACTGGAAAATCAGAGGGCAGGAACTGCTGAACAGCAAAAAGTCAGATAGCACCACATAGCAGACCCGCCATAAAACGCCCTGAGAAGCCCGTGACGGGCTTTTCTTGTATTATGGGTAGTTTCCTTGCATGAATCCATAAAAGGCGCCTGTAGTGCCATTTACCCCCATTCACTGCCAGAGCCGTGAGCGCAGCGAACTGAATGTCACGAAAAAGACAGCGACTCAGGTGCCTGATGGTCGGAGACAAAAGGAATATTCAGCGATTTGCCCGAGCTTGCGAGGGTGCTACTTAAGCCTTTAGGGTTTTAAGGTCTGTTTTGTAGAGGAGCAAACAGCGTTTGCGACATCCTTTTGTAATACTGCGGAACTGACTAAAGTAGTGAGTTATACACAGGGCTGGGATCTATTCTTTTTATCTTTTTTTATTCTTTCTTTATTCTATAAATTATAACCACTTGAATATAAACAAAAAAAACACACAAAGGTCTAGCGGAATTTACAGAGGGTCTAGCAGAATTTACAAGTTTTCCAGCAAAGGTCTAGCAGAATTTACAGATACCCACAACTCAAAGGAAAAGGACTAGTAATTATCATTGACTAGCCCATCTCAATTGGTATAGTGATTAAAATCACCTAGACCAATTGAGATGTATGTCTGAATTAGTTGTTTTCAAAGCAAATGAACTAGCGATTAGTCGCTATGACTTAACGGAGCATGAAACCAAGCTAATTTTATGCTGTGTGGCACTACTCAACCCCACGATTGAAAACCCTACAAGGAAAGAACGGACGGTATCGTTCACTTATAACCAATACGCTCAGATGATGAACATCAGTAGGGAAAATGCTTATGGTGTATTAGCTAAAGCAACCAGAGAGCTGATGACGAGAACTGTGGAAATCAGGAATCCTTTGGTTAAAGGCTTTGAGATTTTCCAGTGGACAAACTATGCCAAGTTCTCAAGCGAAAAATTAGAATTAGTTTTTAGTGAAGAGATATTGCCTTATCTTTTCCAGTTAAAAAAATTCATAAAATATAATCTGGAACATGTTAAGTCTTTTGAAAACAAATACTCTATGAGGATTTATGAGTGGTTATTAAAAGAACTAACACAAAAGAAAACTCACAAGGCAAATATAGAGATTAGCCTTGATGAATTTAAGTTCATGTTAATGCTTGAAAATAACTACCATGAGTTTAAAAGGCTTAACCAATGGGTTTTGAAACCAATAAGTAAAGATTTAAACACTTACAGCAATATGAAATTGGTGGTTGATAAGCGAGGCCGCCCGACTGATACGTTGATTTTCCAAGTTGAACTAGATAGACAAATGGATCTCGTAACCGAACTTGAGAACAACCAGATAAAAATGAATGGTGACAAAATACCAACAACCATTACATCAGATTCCTACCTACATAACGGACTAAGAAAAACACTACACGATGCTTTAACTGCAAAAATTCAGCTCACCAGTTTTGAGGCAAAATTTTTGAGTGACATGCAAAGTAAGTATGATCTCAATGGTTCGTTCTCATGGCTCACGCAAAAACAACGAACCACACTAGAGAACATACTGGCTAAATACGGAAGGATCTGAGGTTCTTATGGCTCTTGTATCTATCAGTGAAGCATCAAGACTAACAAACAAAAGTAGAACAACTGTTCACCGTTACATATCAAAGGGAAAACTGTCCATATATGCACAGATGAAAACGGTGTAAAAAAGATAGATACATCAGAGCTTTTACGAGTTTTTGGTGCATTCAAAGCTGTTCACCATGAACAGATCGACAATGTAACAGATGAACAGCATGTAACACCTAATAGAACAGGTGAAACCAGTAAAACAAAGCAACTAGAACATGAAATTGAACACCTGAGACAACTTGTTACAGCTCAACAGTCACACATAGACAGCCTGAAACAGGCGATGCTGCTTATCGAATCAAAGCTGCCGACAACACGGGAGCCAGTGACGCCTCCCGTGGGGAAAAAATCATGGCAATTCTGGAAGAAATAGCGCTTTCAGCCGGCAAACCTGAAGCCGGATCTGCGATTCTGATAACAAACTAGCAACACCAGAACAGCCCGTTTGCGGGCAGCAAAACCCGTGGGAATTAATTCCCCTGCTCGCGCAGGCTGGGTGCCAAGCTCTCGGGTAACATCAAGGCCCGATCCTTGGAGCCCTTGCCCTCCCGCACGATGATCGTGCCGTGATCGAAATCCAGATCCTTGACCCGCAGTTGCAAACCCTCACTGATCCGCATGCCCGTTCCATACAGAAGCTGGGCGAACAAACGATGCTCGCCTTCCAGAAAACCGAGGATGCGAACCACTTCATCCGGGGTCAGCACCACCGGCAAGCGCCGCGACGGCCGAGGTCTTCCGATCTCCTGAAGCCAGGGCAGATCCGTGCACAGCACCTTGCCGTAGAAGAACAGCAAGGCCGCCAATGCCTGACGATGCGTGGAGACCGAAACCTTGCGCTCGTTCGCCAGCCAGGACAGAAATGCCTCGACTTCGCTGCTGCCCAAGGTTGCCGGGTGACGCACACCGTGGAAACGGATGAAGGCACGAACCCAGTGGACATAAGCCTGTTCGGTTCGTAAGCTGTAATGCAAGTAGCGTATGCGCTCACGCAACTGGTCCAGAACCTTGACCGAACGCAGCGGTGGTAACGGCGCAGTGGCGGTTTTCATGGCTTGTTATGACTGTTTTTTTGGGGTACAGTCTATGCCTCGGGCATCCAAGCAGCAAGCGCGTTACGCCGTGGGTCGATGTTTGATGTTATGGAGCAGCAACGATGTTACGCAGCAGGGCAGTCGCCCTAAAACAAAGTTAAACATCATGAGGGAAGCGGTGATCGCCGAAGTATCGACTCAACTATCAGAGGTAGTTGGCGTCATCGAGCGCCATCTCGAACCGACGTTGCTGGCCGTACATTTGTACGGCTCCGCAGTGGATGGCGGCCTGAAGCCACACAGTGATATTGATTTGCTGGTTACGGTGACCGTAAGGCTTGATGAAACAACGCGGCGAGCTTTGATCAACGACCTTTTGGAAACTTCGGCTTCCCCTGGAGAGAGCGAGATTCTCCGCGCTGTAGAAGTCACCATTGTTGTGCACGACGACATCATTCCGTGGCGTTATCCAGCTAAGCGCGAACTGCAATTTGGAGAATGGCAGCGCAATGACATTCTTGCAGGTATCTTCGAGCCAGCCACGATCGACATTGATCTGGCTATCTTGCTGACAAAAGCAAGAGAACATAGCGTTGCCTTGGTAGGTCCAGCGGCGGAGGAACTCTTTGATCCGGTTCCTGAACAGGATCTATTTGAGGCGCTAAATGAAACCTTAACGCTATGGAACTCGCCGCCCGACTGGGCTGGCGATGAGCGAAATGTAGTGCTTACGTTGTCCCGCATTTGGTACAGCGCAGTAACCGGCAAAATCGCGCCGAAGGATGTCGCTGCCGACTGGGCAATGGAGCGCCTGCCGGCCCAGTATCAGCCCGTCATACTTGAAGCTAGACAGGCTTATCTTGGACAAGAAGAAGATCGCTTGGCCTCGCGCGCAGATCAGTTGGAAGAATTTGTCCACTACGTGAAAGGCGAGATCACCAAGGTAGTCGGCAAATAATGTCTAACAATTCGTTCAAGCCGACGCCGCTTCGCGGCGCGGCTTAACTCAAGCGTTAGATGCACTAAGCACATAATTGCTCACAGCCAAACTATCAGGTCAAGTCTGCTTTTATTATTTTTAAGCGTGCATAATAAGCCCTACACAAATTGGGAGATATATCATGAAAGGCTGGCTTTTTCTTGTTATCGCAATAGTTGGCGAAGTAATCGCAACATCCGCATTAAAATCTAGCGAGGGCTTTACT |

# Supplemental table 5: Sequences of inserts used for cloning

| **Construct** | **DNA sequence** |
| --- | --- |
| YPet_cI 101-229 P158T A152T K192A *Not*I/*Xba*I | ACCCGGCATGACAGGAGGCGGCCGCATCATATGTCTAAAGGTGAAGAATTATTCACTGGTGTTGTCCCAATTTTGGTTGAATTAGATGGTGATGTTAATGGTCACAAATTTTCTGTCTCCGGTGAAGGTGAAGGTGATGCTACGTACGGTAAATTGACCTTAAAATTACTCTGTACTACTGGTAAATTGCCAGTTCCTTGGCCAACCTTAGTCACTACTTTAGGTTATGGTGTTCAATGTTTTGCTAGATACCCAGATCACATGAAACAACATGACTTTTTCAAGTCTGCCATGCCAGAAGGTTATGTTCAAGAAAGAACTATTTTTTTCAAAGATGACGGTAACTACAAGACCAGAGCTGAAGTCAAGTTTGAAGGTGATACCTTAGTTAATAGAATCGAATTAAAAGGTATTGATTTTAAAGAAGATGGTAACATTTTAGGTCACAAATTGGAATACAACTATAACTCTCACAATGTTTACATCACTGCTGACAAACAAAAGAATGGTATCAAAGCTAACTTCAAAATTAGACACAACATTGAAGATGGTGGTGTTCAATTAGCTGACCATTATCAACAAAATACTCCAATTGGTGATGGTCCAGTCTTGTTACCAGACAACCATTACTTATCCTATCAATCTGCCTTATTCAAAGATCCAAACGAAAAGAGAGACCACATGGTCTTGTTAGAATTTTTGACTGCTGCTGGTATTACCGAGGGTATGAATGAATTGTACAAAGAGCTCGCTGCAGGTGGCGGCGGCGGCTCCGGCAGCCATATGTATGAGTACCCTGTTTTTTCTCATGTTCAGGCAGGGATGTTCTCACCTGAGCTTAGAACCTTTACCAAAGGTGATGCGGAGAGATGGGTAAGCACAACCAAAAAAGCCAGTGATTCTGCATTCTGGCTTGAGGTTGAAGGTAATTCCATGACCACACCAACAGGCTCCAAGACAAGCTTTCCTGACGGAATGTTAATTCTCGTTGACCCTGAGCAGGCTGTTGAGCCAGGTGATTTCTGCATAGCCAGACTTGGGGGTGATGAGTTTACCTTCGCGAAACTGATCAGGGATAGCGGTCAGGTGTTTTTACAACCACTAAACCCACAGTACCCAATGATCCCATGCAATGAGAGTTGTTCCGTTGTGGGGAAAGTTATCGCTAGTCAGTGAGGGCCCCCCTTTCGTCTTCAAGAATTCAGTTCTAGAGCGGCCGCCAC |
| *mcI* *Not*I/*Apa*I insert for pBAD-mCI for live cell imaging | ACCCGGCATGACAGGAGGCGGCCGCATCATATGTATGAGTACCCTGTTTTTTCTCATGTTCAGGCAGGGATGTTCTCACCTGAGCTTAGAACCTTTACCAAAGGTGATGCGGAGAGATGGGTAAGCACAACCAAAAAAGCCAGTGATTCTGCATTCTGGCTTGAGGTTGAAGGTAATTCCATGACCACACCAACAGGCTCCAAGACAAGCTTTCCTGACGGAATGTTAATTCTCGTTGACCCTGAGCAGGCTGTTGAGCCAGGTGATTTCTGCATAGCCAGACTTGGGGGTGATGAGTTTACCTTCGCGAAACTGATCAGGGATAGCGGTCAGGTGTTTTTACAACCACTAAACCCACAGTACCCAATGATCCCATGCAATGAGAGTTGTTCCGTTGTGGGGAAAGTTATCGCTAGTCAGTGAGGGCCCCCCTTTCGTCTTCAAGAATTCAGTTCTAGAGCGGCCGCCAC |
| *mcI* *Nde*I/*Eco*RI insert for pETMCSI | ACCCGGCATGACAGGAGGCGGCCGCATCATATGTATGAGTACCCTGTTTTTTCTCATGTTCAGGCAGGGATGTTCTCACCTGAGCTTAGAACCTTTACCAAAGGTGATGCGGAGAGATGGGTAAGCACAACCAAAAAAGCCAGTGATTCTGCATTCTGGCTTGAGGTTGAAGGTAATTCCATGACCACACCAACAGGCTCCAAGACAAGCTTTCCTGACGGAATGTTAATTCTCGTTGACCCTGAGCAGGCTGTTGAGCCAGGTGATTTCTGCATAGCCAGACTTGGGGGTGATGAGTTTACCTTCGCGAAACTGATCAGGGATAGCGGTCAGGTGTTTTTACAACCACTAAACCCACAGTACCCAATGATCCCATGCAATGAGAGTTGTTCCGTTGTGGGGAAAGTTATCGCTAGTCAGTGATAAGGGCCCCCCTTTCGTCTTCAAGAATTCAGTTCTAGAGCGGCCGCCAC |
| *YPet-mcI* *Nde*I/*Eco*RI insert for pETMCSI | ACCCGGCATGACAGGAGGCGGCCGCATCATATGTCTAAAGGTGAAGAATTATTCACTGGTGTTGTCCCAATTTTGGTTGAATTAGATGGTGATGTTAATGGTCACAAATTTTCTGTCTCCGGTGAAGGTGAAGGTGATGCTACGTACGGTAAATTGACCTTAAAATTACTCTGTACTACTGGTAAATTGCCAGTTCCTTGGCCAACCTTAGTCACTACTTTAGGTTATGGTGTTCAATGTTTTGCTAGATACCCAGATCACATGAAACAACATGACTTTTTCAAGTCTGCCATGCCAGAAGGTTATGTTCAAGAAAGAACTATTTTTTTCAAAGATGACGGTAACTACAAGACCAGAGCTGAAGTCAAGTTTGAAGGTGATACCTTAGTTAATAGAATCGAATTAAAAGGTATTGATTTTAAAGAAGATGGTAACATTTTAGGTCACAAATTGGAATACAACTATAACTCTCACAATGTTTACATCACTGCTGACAAACAAAAGAATGGTATCAAAGCTAACTTCAAAATTAGACACAACATTGAAGATGGTGGTGTTCAATTAGCTGACCATTATCAACAAAATACTCCAATTGGTGATGGTCCAGTCTTGTTACCAGACAACCATTACTTATCCTATCAATCTGCCTTATTCAAAGATCCAAACGAAAAGAGAGACCACATGGTCTTGTTAGAATTTTTGACTGCTGCTGGTATTACCGAGGGTATGAATGAATTGTACAAAGAGCTCGCTGCAGGTGGCGGCGGCGGCTCCGGCAGCCACATGTATGAGTACCCTGTTTTTTCTCATGTTCAGGCAGGGATGTTCTCACCTGAGCTTAGAACCTTTACCAAAGGTGATGCGGAGAGATGGGTAAGCACAACCAAAAAAGCCAGTGATTCTGCATTCTGGCTTGAGGTTGAAGGTAATTCCATGACCACACCAACAGGCTCCAAGACAAGCTTTCCTGACGGAATGTTAATTCTCGTTGACCCTGAGCAGGCTGTTGAGCCAGGTGATTTCTGCATAGCCAGACTTGGGGGTGATGAGTTTACCTTCGCGAAACTGATCAGGGATAGCGGTCAGGTGTTTTTACAACCACTAAACCCACAGTACCCAATGATCCCATGCAATGAGAGTTGTTCCGTTGTGGGGAAAGTTATCGCTAGTCAGTGATAAGGGCCCCCCTTTCGTCTTCAAGAATTCAGTTCTAGAGCGGCCGCCAC |
| *PAmCherry-mcI* insert for pND706 (*Nde*I/*Eco*RI) | ACCCGGCATGACAGGAGGCGGCCGCATCATATGGTGAGCAAGGGCGAGGAGGATAACATGGCCATCATTAAGGAGTTCATGCGCTTCAAGGTGCACATGGAGGGGTCCGTGAACGGCCACGTGTTCGAGATCGAGGGCGAGGGCGAGGGCCGCCCCTACGAGGGCACCCAGACCGCCAAGCTGAAGGTGACCAAGGGTGGCCCCCTGCCCTTCACCTGGGACATCCTGTCCCCTCAATTCATGTACGGCTCCAATGCCTACGTGAAGCACCCCGCCGACATCCCCGACTACTTTAAGCTGTCCTTCCCCGAGGGCTTCAAGTGGGAGCGCGTGATGAAATTCGAGGACGGCGGCGTGGTGACCGTGACCCAGGACTCCTCCCTGCAGGACGGTGAGTTCATCTACAAGGTGAAGCTGCGCGGCACCAACTTCCCCTCCGACGGCCCCGTAATGCAGAAGAAGACCATGGGCTGGGAGGCCCTCTCCGAGCGGATGTACCCCGAGGACGGCGCCCTGAAGGGCGAGGTCAAGCCGAGAGTGAAGCTGAAGGACGGCGGCCACTACGACGCTGAGGTCAAGACCACCTACAAGGCCAAGAAGCCCGTGCAGCTGCCCGGCGCCTACAACGTCAACCGCAAGTTGGACATCACCTCACACAACGAGGACTACACCATCGTGGAACAGTACGAACGTGCCGAGGGCCGCCACTCCACCGGCGGCATGGACGAGCTGTACAAGGAGCTCGCTGCAGGTGGCGGCGGCGGCTCCGGCAGCCACATGTATGAGTACCCTGTTTTTTCTCATGTTCAGGCAGGGATGTTCTCACCTGAGCTTAGAACCTTTACCAAAGGTGATGCGGAGAGATGGGTAAGCACAACCAAAAAAGCCAGTGATTCTGCATTCTGGCTTGAGGTTGAAGGTAATTCCATGACCACACCAACAGGCTCCAAGACAAGCTTTCCTGACGGAATGTTAATTCTCGTTGACCCTGAGCAGGCTGTTGAGCCAGGTGATTTCTGCATAGCCAGACTTGGGGGTGATGAGTTTACCTTCGCGAAACTGATCAGGGATAGCGGTCAGGTGTTTTTACAACCACTAAACCCACAGTACCCAATGATCCCATGCAATGAGAGTTGTTCCGTTGTGGGGAAAGTTATCGCTAGTCAGTGAGGGCCCCCCTTTCGTCTTCA |
| *recA* geneblock | CCCGGCATGACAGCTCGAGAGTAACATatggctatcgacgaaaacaaacagaaagcgttggcggcagcactgggccagattgagaaacaatttggtaaaggctccatcatgcgcctgggtgaagaccgttccatggatgtggaaaccatctctaccggttcgctttcactggatatcgcgcttggggcaggtggtctgccgatgggccgtatcgtcgaaatctacggaccggaatcttccggtaaaaccacgctgacgctgcaggtgatcgccgcagcgcagcgtgaaggtaaaacctgtgcgtttatcgatgctgaacacgcgctggacccaatctacgcacgtaaactgggcgtcgatatcgacaacctgctgtgctcccagccggacaccggcgagcaggcactggaaatctgtgacgccctggcgcgttctggcgcagtagacgttatcgtcgttgactccgtggcggcactgacgccgaaagcggaaatcgaaggcgaaatcggcgactctcacatgggccttgcggcacgtatgatgagccaggcgatgcgtaagctggcgggtaacctgaagcagtccaacacgctgctgatcttcatcaaccagatccgtatgaaaattggtgtgatgttcggtaacccggaaaccactaccggtggtaacgcgctgaaattctacgcctctgttcgtctcgacatccgtcgtatcggcgcggtgaaagagggcgaaaacgtggtgggtagcgaaacccgcgtgaaagtggtgaagaacaaaatcgctgcgccgtttaaacaggctgaattccagatcctctacggcgaaggtatcaacttctacggcgaactggttgacctgggcgtaaaagagaagctgatcgagaaagcaggcgcgtggtacagctacaaaggtgagaagatcggtcagggtaaagcgaatgcgactgcctggctgaaagataacccggaaaccgcgaaagagatcgagaagaaagtacgtgagttgctgctgagcaacccgaactcaacgccggatttctctgtagatgatagcgaaggcgtagcagaaactaacgaagatttttaaTCTAGAGCGGCCGCCACC |
